# The Cochrane risk of bias assessment tool 2 (RoB 2) versus the original RoB: A perspective on the pros and cons

**DOI:** 10.1002/hsr2.2165

**Published:** 2024-06-03

**Authors:** Seyed Aria Nejadghaderi, Maryam Balibegloo, Nima Rezaei

**Affiliations:** ^1^ HIV/STI Surveillance Research Center, and WHO Collaborating Center for HIV Surveillance, Institute for Futures Studies in Health Kerman University of Medical Sciences Kerman Iran; ^2^ Cancer Immunology Project (CIP) Universal Scientific Education and Research Network (USERN) Tehran Iran; ^3^ Systematic Review and Meta‐analysis Expert Group (SRMEG) Universal Scientific Education and Research Network (USERN) Tehran Iran; ^4^ Cancer Immunology Project (CIP) Universal Scientific Education and Research Network (USERN) Chicago Illinois USA; ^5^ Network of Immunity in Infection, Malignancy and Autoimmunity (NIIMA) Universal Scientific Education and Research Network (USERN) Tehran Iran; ^6^ Research Center for Immunodeficiencies, Children's Medical Center Tehran University of Medical Sciences Tehran Iran; ^7^ Department of Immunology, School of Medicine Tehran University of Medical Sciences Tehran Iran

**Keywords:** Cochrane, quality assessment, risk of bias, risk of bias assessment tool

## Abstract

**Background and Aims:**

Critical appraisal or risk of bias assessment is a fundamental part of systematic reviews that clarifies the degree to which included research articles are qualified and reliable. Version 2 of the Cochrane tool for assessing the risk of bias in randomized trials (RoB 2), the updated version of the first tool, was released in 2019. Here, we have compared these two versions of Cochrane risk of bias assessment tools and highlighted the pros and cons of RoB 2.

**Methods:**

Statistical analysis and methodology is not applicable to this article as no new data were created or analyzed in this study.

**Results:**

The overall approach in RoB 2 is that by answering some signaling questions after the specification of results, effects of interest, and sources of information, an overall judgment for the quality of each study is reached. Accordingly, in the original version of the Cochrane RoB tool, the judgment can be in three different conclusions, including low, unclear, and high risk of bias. The most prominent difference in bias domains is the removal of “other bias” domain being replaced by “overall bias” judgment. Also, the most common presentation types of Cochrane risk of bias assessments are the “summary” and “graph” which are generated by Review Manager, web‐based applications, or packages in R software.

**Conclusion:**

The RoB 2 tool, compared to the original RoB, has improved and is the recommended version by the Cochrane Collaboration for quality assessment of randomized controlled trials. It is recommended to consider funding source, duration of follow‐up, declaration of data availability, the status of baseline characteristics between groups, and sample size calculation methods in further revisions of the Cochrane risk of bias assessment tools.

## 1 INTRODUCTION

Critical appraisal or risk of bias assessment is a fundamental part of systematic reviews that clarifies the degree to which included research articles are qualified and reliable.^1^ Various risk of bias assessment tools have been developed such as ROBINS‐I (Risk Of Bias In Non‐randomized Studies‐of Interventions) and MINORS (Methodological Index for Non‐Randomized Studies) tools for non‐randomized interventional studies, AXIS (Appraisal tool for Cross‐Sectional Studies) and Crombie's for cross‐sectional studies, ROBIS (Risk of Bias in Systematic Reviews assessment) tool and AMSTAR 2 (A MeaSurement Tool to Assess systematic Reviews version 2) for systematic reviews, SYRCLE (The SYstematic Review Center for Laboratory animal Experimentation), RoB (Risk of Bias) tool, and CAMARADES (The Collaborative Approach to Meta Analysis and Review of Animal Data from Experimental Studies) tool for animal studies, and QUADAS‐2 (A Revised Tool for the Quality Assessment of Diagnostic Accuracy Studies) tool for diagnostic accuracy studies.^2^ Furthermore, some tools like CASP (Critical Appraisal Skills Programme) checklist, NIH (National Institutes of Health) quality assessment tool, JBI (Joanna Briggs Institute) critical appraisal checklist, and SIGN (Scottish Intercollegiate Guidelines Network) methodology checklist are applicable to different types of clinical research.^2^ Version 2 of the Cochrane tool for assessing the risk of bias in randomized trials (RoB 2), the updated version of the first tool,^3^ was released in 2019.^4^

In 2019, Sterne et al. published a methodological study and introduced the Cochrane RoB 2, its development, changes, and improvement in comparison to the previous version, and described each domain and how to interpret and assess study quality using this new quality assessment tool.^4^ Also, the differences between the original RoB and version 2 of Cochrane RoB assessment tools have been mentioned in the Cochrane Handbook of systematic reviews of interventions and previous studies.^1^, ^5^ However, the implications of the results for researchers and methodologists and how the changes can lead to the production of high‐quality systematic reviews were not mentioned. In addition, some domains that were omitted from the original version of RoB assessment were not analyzed in depth in the previous publications. Therefore, herein, we aimed to compare these two versions of Cochrane risk of bias assessment tools and highlight the pros and cons of RoB2 compared to the previous original version of the Cochrane RoB assessment tool (i.e., RoB 1). Moreover, we provide some suggestions and considerations that can be taken into account in the development of the next versions of RoB assessment tools for clinical trials.

## 2 HOW ROB2 IS DIFFERENT FROM ROB1

### 2.1 Included domains

There are differences in bias domains that are included in RoB 2 and the original RoB tool. In the original RoB tool, six bias domains (and source[s] of bias) are assessed, including selection bias, performance bias, detection bias, attrition bias, reporting bias, and “other bias.” For example, the original RoB tool had the following judgment supports for each bias domain: (1) selection bias: Description of the allocation sequence method and the concealment methods of allocation sequence; (2) performance bias: Description of the measurements implemented for blinding of participants and researchers; (3) Detection bias: Description of blinding outcome assessment; (4) Attrition bias: Description of how complete the data for each outcome of interest are presented; (5) Reporting bias: Have the authors only reported selected data that are significant?; (6) Other bias: other potential biases not covered in previous domains.^3^ In the RoB 2 tool, there are five bias domains, including (1) bias due to the randomization process, (2) deviation from intended intervention, (3) missing outcome data, (4) measurement of outcomes, (5) selection of the reported result, and “overall risk of bias” judgment. For instance, the following signaling questions are used to determine the risk of bias for each domain: (1) “Was the allocation sequence random?” and “Was the allocation sequence concealed until participants were enrolled and assigned to interventions?”; (2) “Were participants aware of their assigned intervention during the trial?” and “Were carers and people delivering the interventions aware of participants' assigned intervention during the trial?”; (3) “Were data for this outcome available for all, or nearly all, participants randomized?”; (4) “Was the method of measuring the outcome inappropriate?” and “Could measurement or ascertainment of the outcome have differed between intervention groups?”; and (5) “Were the data that produced this result analyzed in accordance with a prespecified analysis plan that was finalized before unblinded outcome data were available for analysis?”.^4^

The most prominent difference in bias domains is that the “other bias” domain is removed in RoB 2 and instead, an “overall bias” judgment has been added based on the status of the five bias domains. Despite some explanations like “state any important concerns about bias not covered in the other domains in the tool” or “bias due to problems not covered elsewhere,” in our opinion, the “other bias” domain is the most ambiguous domain in the original version of RoB tool. The study conducted by Babic et al. on 768 Cochrane reviews which included 11,369 randomized controlled trials (RCTs) showed that 87% of reviews reported “other bias” domain and there were 5,‐762 types of explanations which were categorized into 31 groups.^6^ Some of the items listed in this domain such as outcome measures, attrition, blinding, selection and randomization, reporting, and publication biases were assessed in other domains of this tool as well, or they were evaluated by other methods. Nevertheless, some items are missing in the RoB 2 tool.

### 2.2 Overall risk of bias judgment

The overall approach in RoB2 is that by answering some signaling questions after the specification of results, effects of interest, and sources of information, an overall judgment for the quality of that study is reached.^4^ However, in the original version of the Cochrane RoB tool, the judgment can be in three different groups, including low, unclear, and high risk based on provided support for judgment.^3^ The presence of signaling questions in RoB2, in five levels, including yes, probably yes, no, probably no, and no information, which leads to high risk, low risk, and some concerns for each domain can increase the precision of this revised version compared to the original version of RoB tool.

### 2.3 Presentation of results

**Figure 1:**
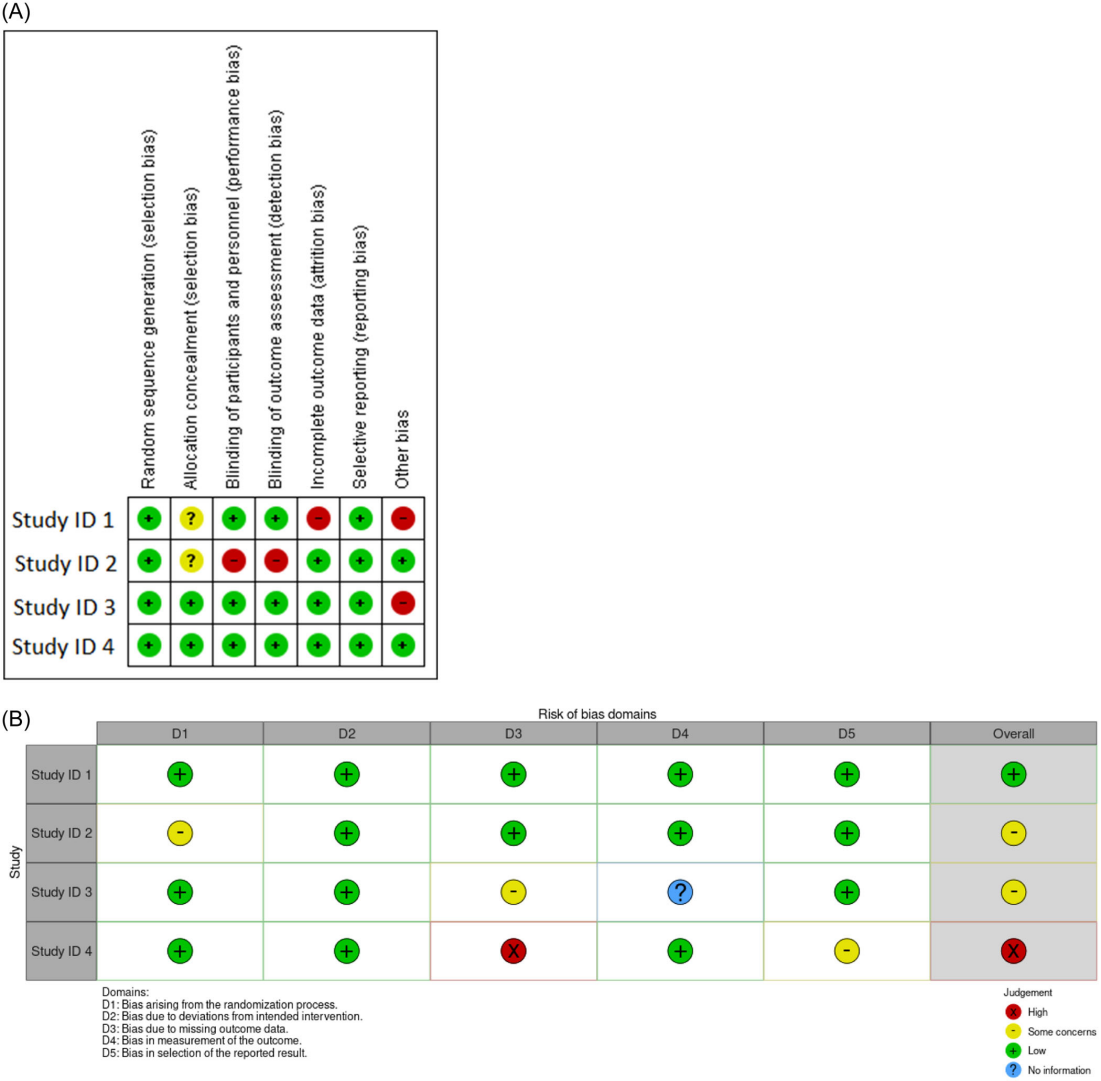
Summary of risk of bias assessment for four samples included studies using Cochrane risk of bias assessments tool version 1 (A) and version 2 (RoB 2) (B). “+”: low risk of bias; “?”: unclear risk of bias; “−”: high risk of bias for version 1.

The most common presentation types of Cochrane risk of bias assessments are the “summary” and “graph” which are generated by Review Manager (RevMan) (Copenhagen: The Nordic Cochrane Centre, The Cochrane Collaboration, 2014) or RevMan Web application. Also, other programs were developed to facilitate the generation of results of RoB such as Risk‐of‐bias VISualization (robvis) which is a package for R programming software, or Shiny web app.^7^ Also, the web app designed version of the RoB2 is available at https://www.riskofbias.info/welcome/robvis-visualization-tool and can be used for the creation of traffic light plots and weighted bar plots for quality assessment. There were no remarkable changes in presenting the results between RoB 2 (Figure 1A) and the original version of the RoB assessment tool (Figure 1B).

## 3 LIMITATIONS AND IMPROVEMENTS OF ROB 2

The RoB 2 improved the quality assessment of randomized clinical trials in several ways. Contrary to the original version of the Cochrane RoB tool which was based on the authors' judgments and used quotes from the papers to support the judgments, the RoB 2 used a more comprehensive and structured framework for quality assessment using the signaling questions. This approach will help reviewers for quality assessment, especially for complex RCTs.

Despite the RoB 1 that has one guidance and iteration, the RoB 2 includes other supplements for parallel, cluster‐randomized, and cross‐over designs of RCTs. It can increase the precision and facility of risk of bias assessment. However, there is still a lack of some supplements for other types of clinical trials like clinical trials without a control arm or RCTs with factorial design.

Both versions of the Cochrane RoB assessment tools use another option for RoB that cannot be categorized into low or high RoB. In this regard, the original version uses “unclear” and the RoB 2 uses “some concerns.” Nevertheless, the RoB 2 improved the criteria for judgment on how to reach an overall judgment about the RoB using the signaling questions and algorithms. Also, the RoB 2 is focused on the results instead of outcomes.^5^

On the other hand, one of the main limitations of the RoB 2 is that it is complex and needs trained individuals to use this tool. One of the limitations of the RoB2 is that it takes more time to complete each study. A systematic review of 113 studies showed an average time of 358 min per article.^8^ While this time for the original RoB tool was between 10 and 60 min per article.^9^ Two different studies assessing the RoB 2 showed no significant inter‐rater reliability (IRR: −0.15) and slightly higher IRR (IRR: 0.16) for overall judgment.^10^, ^11^ Nevertheless, there was a significant association between the use of RoB 2 and the improved methodological quality of the systematic reviews (*p* = 0.007).^12^ Overall, the RoB 2 is comprehensive but complex and difficult to use even for experts.

In spite of the improvement in the assessment methods in the RoB2, there are still domains that are subjective and there might be doubt and disagreement about the answer to the signaling questions. For example, the results of a comparison study between the two versions of the Cochrane RoB assessment tools showed that the responses to “Were there deviations from the intended intervention that arose because of the trial context?” question are usually “no information” or “probably no” due to no available protocol which leads to overall judgments of “some concerns” or low RoB, respectively and can lead to discrepancy in the findings and reporting of RoB.^13^

## 4 DISCUSSION

In the present article, we briefly discussed the differences between the Cochrane RoB 2 and the original version of the RoB assessment tool. Overall, the RoB 2 assesses five domains and includes an overall RoB judgment domain. While the original RoB tool includes six predefined domains and also has a domain for other types of biases that were not included in other parts (Table 1).^3^, ^4^ In terms of output presentation, RevMan was among the first software which was developed for the presentation of RoB assessment results. Then, other web‐based applications and packages in other software like R were designed.

**Table 1:** Bias domains that are assessed in version 2 and the original version of the Cochrane risk‐of‐bias (RoB) assessment tool for randomized trials and approach to the overall risk of bias judgment or across trials.

Cochrane RoB 2	Original Cochrane RoB
Randomization process bias	Selection bias (including random sequence generation and allocation concealment)
Deviations from intended interventions	Performance bias due to blinding status of participants/researchers
Missing outcome data	Detection bias due to blinding status of outcome assessment
Measurement of the outcome	Attrition bias due to incomplete data
Selection of the reported result	Reporting bias due to selective reporting
Overall bias	Other bias
**General bias judgment**	
Low: low risk of bias for all domains	Low: “Most information is from trials at low risk of bias”
Some concerns: Some concerns in at least one domain, but not to be at high risk of bias for any domain	Unclear: “Most information is from trials at low or unclear risk of bias”
High: High risk of bias in at least one domain for this result, or the study is judged to have some concerns for multiple domains	High: “The proportion of information from trials at high risk of bias is sufficient to affect the interpretation of results”

As we have previously discussed, Cochrane RoB 2 is improved in different aspects like focusing on results, improved guidance for decisions on the overall judgment for each domain, enhanced structure, and facilitation of justification for each domain. However, there are still several limitations that might be considered in the next iterations. One of the changes is omitting the “other bias” domain in RoB 2 compared with RoB 1. The study by Babic et al. on 768 Cochrane reviews showed that baseline characteristics of participants (21.4%), funding (15.6%), sample size (8.1%), reporting (7.6%), and conflicts of interest (5.8%) are the five most frequent categories reported for “other bias” domain in systematic reviews.^6^

There are some debates about reporting funding and conflicts of interest in the systematic reviews. The AMSTAR 2, which is a tool for quality assessment of systematic reviews, includes two domains related to the source of funding (Domain 10: “Did the review authors report on the sources of funding for the studies included in the review?” and Domain 16: “Did the review authors report any potential sources of conflict of interest, including any funding they received for conducting the review?”).^14^ Some experts believe adding a source of funding as a domain for RoB assessment might lead to a decrease in the willingness of industries to collaborate with researchers and RCT design, whereas they agree that it is necessary to report conflicts of interest and to provide improved tools for reporting bias assessment.^15^ In this regard, a Cochrane review that included 75 articles compared favorable outcomes between industry‐funded studies and studies using other funding sources.^16^ It showed that the results are in favor of better efficacy findings in industry‐funded studies than other sources of funding (risk ratio [RR]: 1.27; 95% confidence interval [CI]: 1.17–1.37).^16^ As a result, there can be an “industry bias” that is not assessed in the RoB 2 which might be considered for designing future quality assessment tools.

Protocol registration and deviation from registered protocol were reported in 0.3% and 2.5% of the “other bias” domain in the RoB1, respectively.^6^ The RoB 1 and RoB 2 include the “reporting bias” domain and “bias in the selection of the reported result,” respectively, to assess this outcome. the addition of some specific signaling questions to assess the registration of trial protocol in online registration platforms before the initiation of the study can be a useful method to help better evaluate the RoB of RCTs.

Recently, some other novel assessment tools have been developed for the quality assessment of RCTs. In this regard, Weibel and colleagues generated a research integrity assessment tool to identify problematic RCTs which evaluates six criteria, including ethics approval status, study retraction, prospective trial registration, author group, plausibility of methods, and study results.^17^

Our current study has some limitations and points that should be acknowledged. It is a perspective article that only criticized the advantages and disadvantages of the RoB 2 compared to the original version and provided some factors that need to be considered in future iterations of RoB assessment tools for RCTs. However, it did not collect and report primary original data. Instead, it reported some results of some previous studies to support the findings. It only assessed Cochrane RoB 2 with a focus on the version for parallel study design and compared it with RoB 1, while other quality assessment tools for clinical trials or observational studies were not evaluated in the article. The suggestions for consideration in the development of the next quality assessment tools are based on the previous findings and authors' opinions. Further research, pilot studies, and expert consensus might be necessary to evaluate whether they can be implemented in future RoB tools.

## 5 CONCLUSIONS

The RoB 2 tool, compared to the original RoB, has been improved and is the recommended version by Cochrane for the quality assessment of RCTs.^18^ The judgment process and the conclusion of each study's risk of bias have been changed, while the presentation of the assessment is almost similar. Meanwhile, omitting the “other bias” domain has improved the clarity of RoB 2. Nevertheless, still some improvements need to be implemented such as considering some valuable information addressing the quality of studies. In addition, it is recommended that funding source, duration of follow‐up, declaration of availability of data, the status of baseline characteristics between groups, and methods on sample size calculation be taken into consideration for further revisions of the Cochrane RoB assessment tool.

## AUTHOR CONTRIBUTIONS

**Seyed Aria Nejadghaderi**: Conceptualization; investigation; methodology; writing—original draft; writing—review and editing. **Maryam Balibegloo**: Conceptualization; methodology; resources; writing—original draft; writing—review and editing. **Nima Rezaei**: Conceptualization; methodology; project administration; supervision; writing—review and editing.

## CONFLICT OF INTEREST STATEMENT

The authors declare no conflict of interest.

## TRANSPARENCY STATEMENT

The lead author Nima Rezaei affirms that this manuscript is an honest, accurate, and transparent account of the study being reported; that no important aspects of the study have been omitted; and that any discrepancies from the study as planned (and, if relevant, registered) have been explained.

## Data Availability

Data sharing is not applicable to this article as no new data were created or analyzed in this study.
